# Do Microorganisms in Bathing Water in Guadeloupe (French West Indies) Have Resistance Genes?

**DOI:** 10.3390/antibiotics13010087

**Published:** 2024-01-16

**Authors:** Degrâce Batantou Mabandza, Edlyne Colletin, Christophe Dagot, Isaure Quétel, Sébastien Breurec, Stéphanie Guyomard-Rabenirina

**Affiliations:** 1 Transmission, Reservoir and Diversity of Pathogens Unit, Pasteur Institute of Guadeloupe, 97110 Pointe-à-Pitre, France; 2 University of Limoges, INSERM, CHU Limoges, RESINFIT, U1092, 87000 Limoges, France; 3 Faculty of Medicine Hyacinthe Bastaraud, University of the Antilles, 97110 Pointe-à-Pitre, France; 4 INSERM, Centre for Clinical Investigation 1424, 97110 Pointe-à-Pitre, France; 5 Department of Pathogenesis and Control of Chronic and Emerging Infections, University of Montpellier, INSERM, 34394 Montpellier, France; 6 Laboratory of Clinical Microbiology, University Hospital Centre of Guadeloupe, 971110 Pointe-à-Pitre, France

**Keywords:** *Escherichia coli*, resistome, biofilm, river, pollution

## Abstract

Waterborne faecal contamination is a major public health concern. The main objectives of this study were to investigate faecal contamination and *Escherichia coli* (*E. coli*) antibiotic resistance in recreational fresh water from Guadeloupe and to characterise the microbiome and resistome composition in biofilms from submerged rocks. Significant faecal contamination was observed at 14 freshwater sites. *E. coli* predominated (62%), followed by *Enterobacter cloacae* (11%) and *Acinetobacter* spp. (11%). Of 152 *E. coli* isolated, none produced extended-spectrum beta-lactamases (ESBLs), but 7% showed resistance to streptomycin and 4% to tetracycline. Biofilm resistome analysis revealed clinically significant antibiotic-resistance genes (ARGs), including those coding for resistance to sulfonamides (*sul1*), carbapenems (*bla*_KPC_), and third-generation cephalosporins (*bla*_CTX-M_). Mobile genetic elements (MGEs) (*intI1*, *intI2*, *intI3*) linked to resistance to aminoglycosides, beta-lactams, tetracycline, as well as heavy metal resistance determinants (*copA*, *cusF*, *czcA*, *merA*) conferring resistance to copper, silver, cadmium, and mercury were also detected. Diverse bacterial phyla were found in biofilm samples, of which Proteobacteria, Bacteroidetes, Planctonomycetes, and Cyanobacteria were predominant. Despite the frequent presence of *E. coli* exceeding regulatory standards, the low levels of antibiotic-resistant bacteria in freshwater and of ARGs and MGEs in associated biofilms suggest limited antibiotic resistance in Guadeloupean recreational waters.

## 1. Introduction

Antibiotics are commonly used in human and veterinary medicine to treat or prevent bacterial infections [1,2]; however, the over- and inappropriate use of these drugs has led to a rise in antibiotic resistance in bacteria. Non-antibiotic compounds, such as antibacterial biocides and heavy metals, can also contribute to the rise in antibiotic resistance through co-selection mechanisms [3]. Horizontal gene transfer (HGT), facilitated by mobile genetic elements (MGEs) like integrons, transposons, and plasmids, enables the exchange of resistance-conferring genes to these elements [4]. Antimicrobial resistance (AMR) is now a major public health problem worldwide [5,6,7].

Guadeloupe, a French overseas tropical island in the Caribbean, is a major element in human migration between Europe, the USA, and other Caribbean islands [8]. With its high economic level (https://hdr.undp.org, accessed on 9 March 2023), insularity, small surface area (1436 km^2^), and small population (375,845 inhabitants in 2023), it is a propitious environment for studying reservoirs of antibiotic-resistant isolates and identifying routes of transmission to the human population.

Data on resistance to antibiotics in Guadeloupe are scarce and recent. The island has faced the emergence of nosocomial infections associated with carbapenemase-producing Enterobacteriaceae since 2014 and also a high incidence of nosocomial infections caused by ESBL-producing Enterobacteriaceae [9,10,11,12]. Environmental investigations have focused on the faecal carriage of ESBL-producing Enterobacteriaceae in domestic animals [13,14], farm animals [15], and wildlife [16,17] and also on the transmission of clinical strains to the environment [8,18]. Antibiotic resistance was also studied in effluents from wastewater treatment plants (WWTP) and in surface waters with or without such discharges [18,19]. Although these studies concluded that there is limited circulation of antibiotic-resistant bacteria (ARB) between humans and the environment [8,13,16,18], there are still gaps in understanding the transmission of ARB from the environment to humans.

Guadeloupe, renowned for its beaches and rivers, welcomes large numbers of tourists, with over 815,000 visitors in 2019 [20], and aquatic recreation plays a central role in tourism. Monitoring water quality is therefore essential. The Regional Health Agency has mandated the Pasteur Institute of Guadeloupe to conduct monthly monitoring of the bacteriological quality of all the beaches and rivers of the archipelago on which aquatic recreation is practised. Microbiological water quality control is based on the monitoring of faecal contamination indicators such as *E. coli* and intestinal enterococci [21]. In 2020, 11% of Guadeloupe’s recreational waters did not meet the required quality standards (59% of river monitoring sites), and bathing was prohibited at 3% of the sites [22]. Despite growing concern about faecal pollution of water in Guadeloupe, data on AMR in recreational areas that do not receive WWTP discharges are scarce. The only study, conducted in 2016 [19], revealed limited antibiotic resistance in such water; however, the conclusion was limited to surface waters and did not address the issue of biofilms.

It is now well established that ARB and their resistance determinants can infiltrate surface waters [23,24,25,26] and biofilms [27,28,29]. Biofilms can adhere to a variety of surfaces, including rocks in rivers. Such bacterial communities form a complex extracellular matrix that confers high resistance to antimicrobial treatment. Previous studies have reported the presence of ARB in river biofilms, sediments, and the water column along river systems [30,31,32].

The aim of this exploratory study was to assess the contamination of recreational river surface water and biofilms by antibiotic-resistant coliforms and broaden the understanding of antibiotic resistance on the island of Guadeloupe. The main objective was to characterise antibiotic resistance in 14 rivers known to have poor water quality. In addition, we analysed the microbiome and resistome of biofilms present on rocks at the bottom of three of the most polluted rivers. Surface water contamination by faecal coliforms was estimated by culturing *E. coli*, and the characterisation of phenotypic resistance was limited to this species. Unlike enterococci, antibiotic-resistant *E. coli* can be considered the sentinels of AMR in the environment [33,34]. The abundance of resistance determinants was assessed by Fluidigm polymerase chain reaction (PCR) in biofilms, and the microbiome was characterised by metabarcoding targeting the 16S rRNA gene.

## 2. Results

### 2.1. Enumeration of E. coli in Water Samples

*E. coli* were counted by the “most probable number” (MPN) method. Of 59 river samples, 48 had an MPN of <100 colony-forming units (CFUs)/100 mL, of which 15 had none. The lowest value in 11 samples with MPN values > 100 CFU/100 mL was 110 CFU/100 mL (Grande Rivière du Lamentin) and the highest value was 2508 CFU/100 mL (Corossol). Only six sites had high levels of faecal bacteria, and no *E. coli* were found at Sofaïa (Table 1). During campaign 3, when there was heavy rainfall, *E. coli* levels were >1000 CFU/100 mL at only three sites (Table 1).

### 2.2. Characterisation of Bacterial Isolates from River Water, Antibiotic Susceptibility of E. coli, and Resistance in Biofilms

Blue colonies were confirmed as *E. coli* on chromogenic media by matrix-assisted laser desorption/ionisation mass spectrometry. Other colonies of various colours were selected randomly for the identification of bacterial diversity in bathing water. During the five sampling campaigns, 245 bacteria were identified, the most common species being *E. coli* (*n* = 152, 62%), followed by *Enterobacter cloacae* (*n* = 25, 11%) and *Acinetobacter* spp. (*n* = 25, 11%). Other relevant species (*Citrobacter freundii*, *C. amalonaticus*, *Klebsiella pneumoniae*, *K. variicola*, *K. oxytoca*, *K. ascorbata*, *Aeromonas hydrophila*, *Ae. enteropelogenes*, *Ae. jandaei Pseudomonas putida*, *Serratia marcescens*, *Chromobacterium violaceum*, *Pseudomonas alcalifaciens*, *Providencia alcalifaciens*, *Raoultella ornithinolytica*, and *Cronobacter* spp.) were less abundant.

No *E. coli* strains were detected in the medium with added third-generation cephalosporin. None of the 47 *E. coli* strains tested for antibiotic susceptibility showed resistance, except for streptomycin (*n* = 3, 7% resistance) and tetracycline *(n* = 2, 4% resistance). These results suggest little bacterial resistance in recreational river waters from Guadeloupe. In order to confirm these results, we characterised the resistome of biofilms present in the three most polluted rivers (Corossol, Fond Helliot, and Grande Rivière du Lamentin).

**Figure 1:**
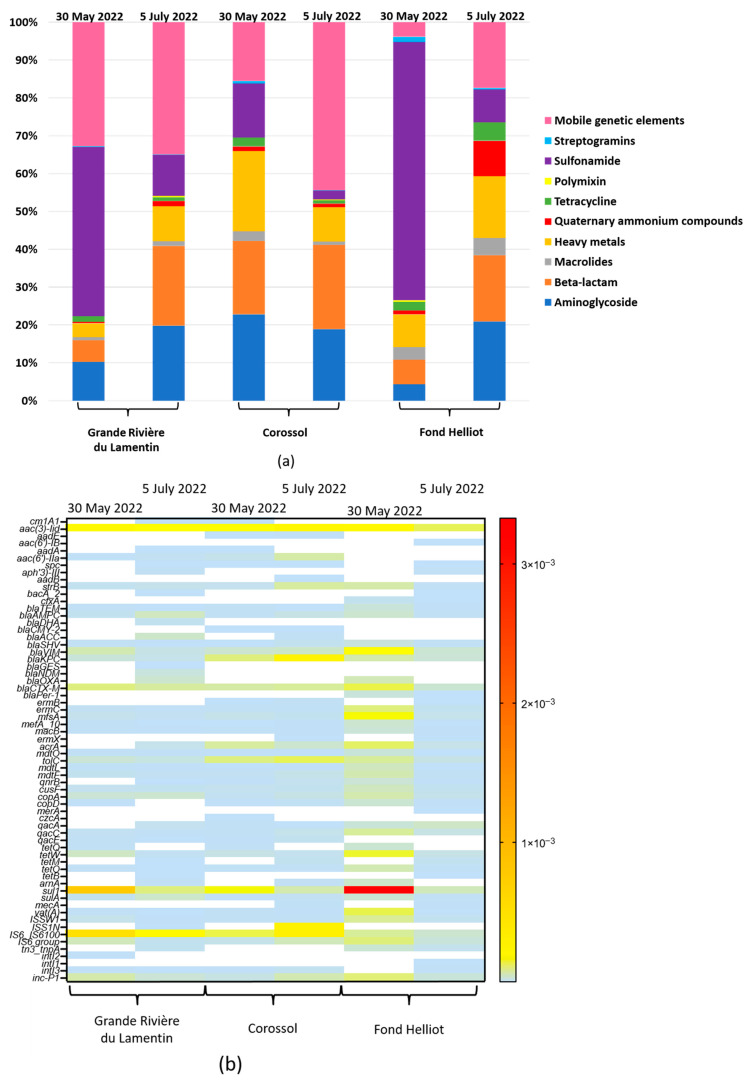
(**a**) Normalised relative abundance of antimicrobial classes (antibiotic-resistance gene [ARG] copies per 16S rRNA gene copy) in six biofilm samples. (**b**) Heat map of the relative abundance of the 64 ARGs detected in the six biofilm samples (ARG copy per 16S rRNA gene copy). White bands represent undetected elements.

In two campaigns, nine categories of clinical resistance genes associated with streptogramin, macrolide, sulfonamide, polymyxin, tetracycline, beta-lactam, aminoglycoside, quaternary ammonium compounds (QAC), and heavy metal resistance were identified. In addition, MGEs were present at all three sites (Figure 1a). The relative abundance of resistance determinants and MGEs differed in the two sampling campaigns, especially at the Corossol and Fond Helliot sites (Figure 1a). Sulphonamide resistance genes were more abundant during the first campaign (30 May 2022) (Figure 1a), while beta-lactam, aminoglycoside, heavy-metal resistance genes, and MGEs were more prevalent in the second campaign (5 July 2022) (Figure 1a). No major difference was observed between the two campaigns for the other resistance determinants, with the exception of tetracycline and QAC resistance genes, which were particularly abundant at the Fond Helliot site during the second campaign (Figure 1a).

A total of 64 subtypes of resistance determinants were identified in the samples analysed, although in small quantities (Figure 1b). The number of ARG subtypes varied from 34 (minimum) to 49 (maximum) per sample (Figure 1b) and the abundance from 3.30 × 10^−7^ to 3.33 × 10^−3^ copies/16S rRNA gene. Similar numbers of ARGs and MGE were detected at all sites. The Fond Helliot site had a higher overall relative abundance than the other sites on 30 May 2022 (Figure 1b). Overall, the signal intensity of resistance determinants and MGEs was more pronounced during the first campaign (Figure 1b), particularly at Grande Rivière du Lamentin and Fond Helliot, while in the second campaign, signal intensity was higher at Corossol. The five most abundant subtypes at all the sites in both campaigns were the resistance genes *sul1*, *aac(3)-Iid*, *bla_KPC_*, *bla_CTX-M_*, and *bla_VIM_* (Figure 1b), although the abundance of *sul1* was greatest at Fond Helliot in the first campaign. The MGEs identified were inc-P1 plasmid, transposon Tn3 (*tnpA*), insertion sequences (*IS6* family, *ISS1*, *ISS1W*), and integrons including *intI1*, *intI2*, and *intI3* (Figure 1b), some of which were ubiquitous, while others were detected only at certain sites. Thus, the analysis of biofilm resistomes from the rivers studied indicated only limited resistance determinants.

### 2.3. Composition of Bacterial Communities in Biofilms

**Figure 2:**
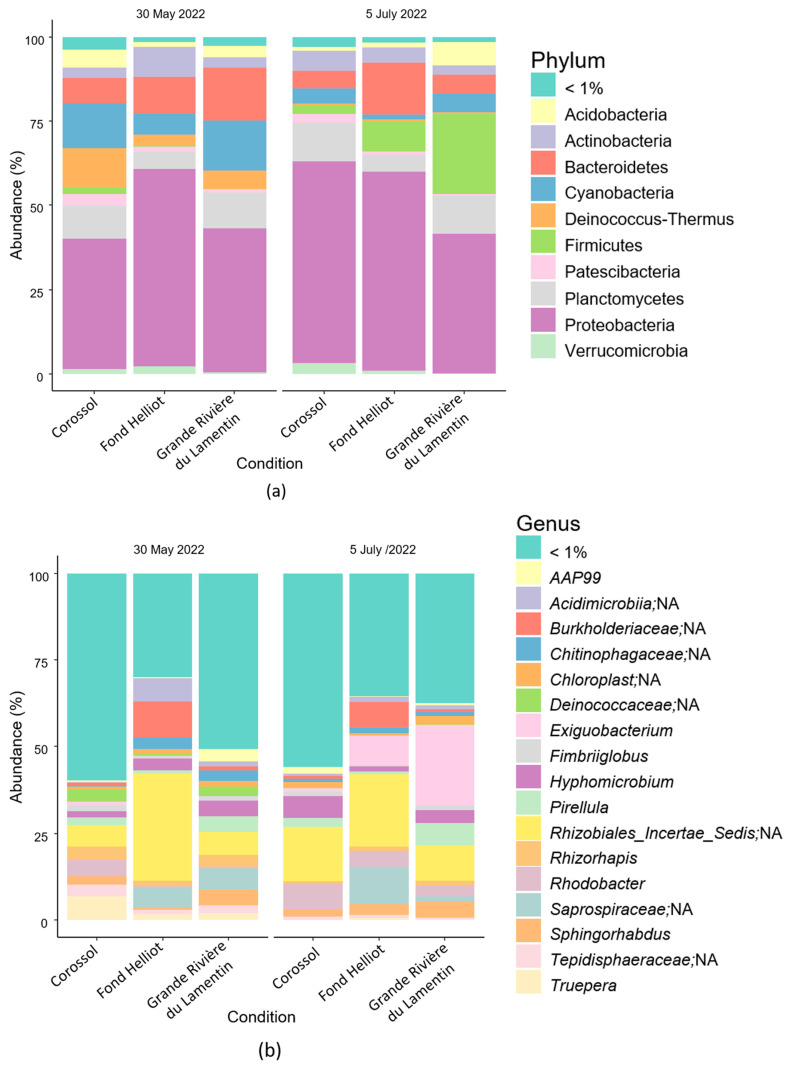
Normalised relative abundance of bacteria at phylum (**a**) and genus (**b**) level in six biofilm samples from the Corossol, Fond Helliot, and Grande Rivière du Lamentin rivers on 30 May 2022 and 5 July 2022. NA means not assigned. For names with NA, the genera have not been identified. We have referred to the last taxonomic assignment found.

After quality filtering, 1,039,932 normalised sequences were obtained from six biofilm samples, i.e., 173,322 sequences per sample. An analysis of the 16S sequencing data indicated the presence of several bacterial communities in river water biofilms. A total of 13,404 amplicon sequence variants was found in the prokaryotic communities. 16S rRNA gene sequencing showed that these bacterial communities comprised 40 phyla, 99 classes, 223 orders, 320 families, and 740 genera. The bacterial communities in biofilms from the Corossol, Fond Helliot, and Grande Rivière du Lamentin rivers were similar at the phylum (Figure 2a) and genus (Figure 2b) levels, despite their geographical separation, although the relative abundance varied. Proteobacteria (50% of all phyla) were the dominant phylum in all samples (Figure 2a). The major phyla at other sampling sites or campaigns were Bacteroidetes (10% of all phyla), Planctomycetes (9% of all phyla), and Cyanobacteria (8% of all phyla) (Figure 2a), and the predominant bacterial genera were *Rhizobiales*, *Burkholderiaceae*, *Exiguobacterium*, and *Saprospiraceae*, accounting for 3.5–15.1% of all genera (Figure 2b).

Variations in taxonomic relative abundance were observed between the two sampling campaigns. For example, the relative abundance of the Cyanobacteria and Deinococcus-Thermus phyla decreased between the first (30 May 2022) and second (5 July 2022) campaign (35% (sum of relative abundances) vs. 11%, 20% vs. 2%, respectively), while the relative abundance of Firmicutes increased (3% vs. 35%), especially at the Fond Helliot and Grande Rivière du Lamentin sites (Figure 2a). At the genus level, *Exiguobacterium* was more abundant at both sites during the second sampling campaign (Figure 2b). *Truepera* and *Deinococcaceae* were detected in low proportions during the first sampling campaign and almost disappeared during the second campaign (Figure 2b). A similar trend was observed for *Saprospiraceae* at the Corossol and Grande Rivière du Lamentin sites (Figure 2b). In contrast, the relative abundance of *Rhodobacter* increased at all sites, from a total relative abundance of 6% in the first campaign to 15% in the second (Figure 2b). Geographical variations were also observed. The *Rhizobiales*, *Burkholderiaceae*, and *Acidimicrobiia* genera were predominant at the Fond Helliot site (Figure 2b).

### 2.4. Analysis of Alpha Diversity

**Figure 3:**
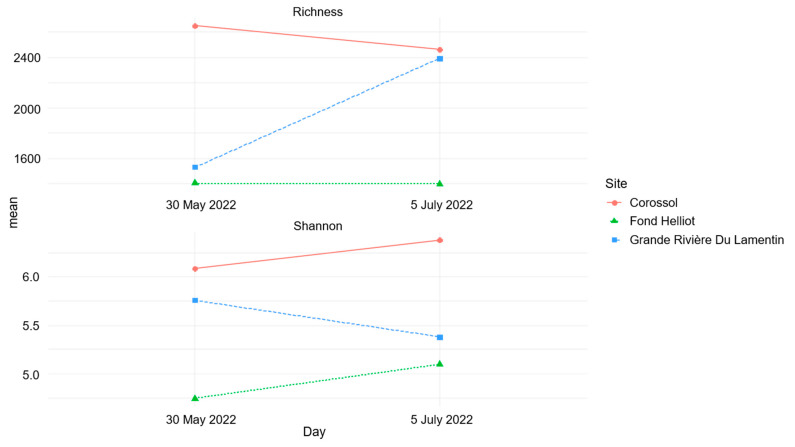
Analysis of alpha diversity. The graph shows the average richness and the Shannon index.

Analysis of variance of the Shannon index showed a significant difference in alpha diversity among the sites (*p* = 0.03) but not among sampling dates (*p* = 0.88). In pairwise comparisons (post hoc Tukey) of similarities, samples from Fond Helliot differed significantly from those from Corossol (*p* = 0.02), while those from Fond Helliot–Grande Rivière du Lamentin and Grande Rivière du Lamentin–Corossol did not (*p* = 0.15 and *p* = 0.14, respectively). These results indicate variations in the specific richness and equitability of communities among sites (Figure 3). The Shannon diversity index, calculated for each sample, confirmed that the Corossol site had the highest bacterial diversity of the three sites (Figure 3).

### 2.5. Analysis of Beta Diversity

**Figure 4:**
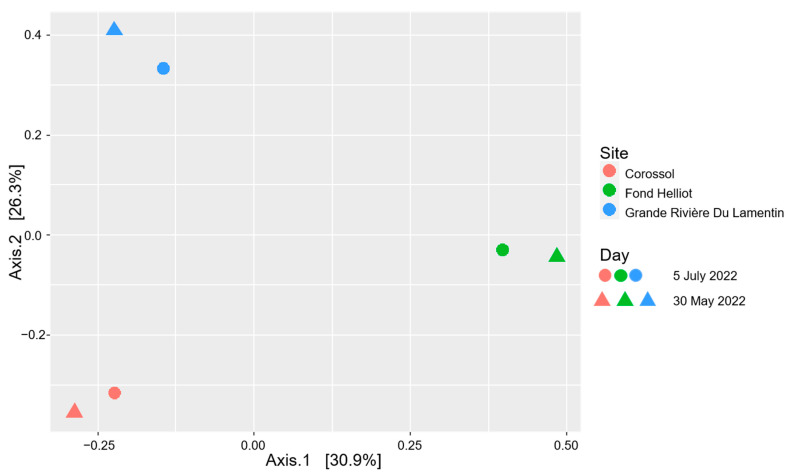
Analysis of beta diversity with the Bray–Curtis index.

Beta diversity analysis (Figure 4) confirmed the similarity of bacterial composition among the sites. No significant differences in the structure of the bacterial community were observed at the different sampling sites (*p* = 0.70) or between the sampling dates (*p* = 0.07).

## 3. Discussion

This study focused on the presence of total and resistant *E. coli* in recreational freshwater unaffected by effluent from wastewater treatment plants, with *E. coli* being found at least once at all study sites except one. Of the 14 sites, 11 had *E. coli* at levels >100 CFU/100 mL, indicating poor water quality and the presence of faecal pollution. During the third campaign, increased *E. coli* levels were found at Bras David, Corossol, and Vallée Verte, indicating that precipitation contributes to soil leaching, with discharge of bacteria into aquatic environments [35,36]. These high *E. coli* loads could be linked to discharges of wildlife faeces, particularly from birds. A low level of antibiotic resistance was found in the *E. coli* isolates studied: no ESBL production was observed and only resistance to streptomycin and tetracycline was detected, due probably to their extensive use in veterinary medicine [37,38]. The results are in line with previous local data indicating a low level of antibiotic resistance in areas with no anthropogenic impact [19]. In contrast, in the Netherlands, ESBL-producing *E. coli* were detected in surface waters not influenced by WWTPs in similar concentrations than those under the influence of WWTPs, indicating the existence of additional ESBL-producing *E. coli* contamination sources [39].

ARGs were detected in biofilms from all river sites, although at low levels, and were associated with resistance to several classes of antibiotics. Interestingly, 28 ARG subtypes were common to all samples, indicating a similar ARG composition. ARGs were associated with resistance to carbapenem and polymyxin, two clinically relevant last-line treatments for life-threatening infections [40,41]. Determinants of resistance to tetracyclines, sulfonamides, beta-lactams, aminoglycosides, and macrolides were also detected. Interestingly, *bla*_CTX-M_ was one of the most frequent ARGs detected, although no bacteria resistant to third-generation cephalosporin were isolated.

The integrons *intI1*, *intI2*, and *intI3* detected here have previously been described as having a clinical origin [4,42,43], with *IntI1* potentially being used as an indicator of environmental resistance [44]. These integrons are mainly linked to horizontal gene transfer among several bacterial species [43]. *intI3* was the only integron identified consistently across all sites and during both sampling periods, and its presence was associated with the soil and freshwater proteobacterial group [42]. We also identified determinants of resistance to heavy metals and disinfectants, which can accelerate the spread of ARGs and antibiotic-resistant bacteria while promoting horizontal gene transfer [45,46,47,48,49]. Although the sample used in this study was relatively small for a resistome study, our results suggest that biofilms in these rivers are not sites of permanent ARG accumulation or establishment. More exhaustive sampling would provide a better understanding of environmental impacts such as the spatial and temporal extent of waterborne bacteria carrying ARGs. Nevertheless, our analysis suggests no permanent risk for human health from antibiotic resistance in Guadeloupe’s recreational waters, including biofilms.

An analysis of the biofilm microbiome characteristics from three freshwater sites showed that the predominant bacterial phyla at our study sites were Proteobacteria, Bacteriodetes, Planctomycetes, and Cyanobacteria, as reported in previous studies but with different abundances [50,51,52,53,54,55]. The predominance of Proteobacteria was highlighted in previous studies investigating surface waters [56,57,58,59,60,61] and biofilms from various environments [51,62,63] through 16S rRNA gene analysis. With Proteobacteria considered prolific surface colonisers [64], previous studies have also revealed their predominance in aquatic ecosystems, where they influence biogeochemical cycles such as those of carbon and nitrogen [55,65,66,67]. This is in agreement with the large amounts of nutrients in our freshwater sites, which favour their proliferation. Bacteroidetes have been associated with biopolymer degradation and contribute to dissolving organic matter [68], while Planctonomycetes have been associated with ammonium regulation [69]. Cyanobacteria, a group of aquatic and photosynthetic bacteria, influence the structure and productivity of microbial communities [70]. The *Rhizobiales* and *Burkholderiales* genera were the most abundant, indicating oligotrophic or mesotrophic conditions [71,72]. The genus *Saprospiraceae*, which was also abundant, is commonly associated with wastewater and sewage sludge [73,74]. It is also found in various marine and freshwater environments [74], where it can hydrolyse and use complex organic carbon compounds [75]. Although the residence time of submerged rocks for river biofilm formation was the same in both sampling campaigns, variations in the relative abundances of phyla and genera were observed within the same site. The decrease or increase in intra-river relative abundance of specific taxa between the two sampling periods suggests a sensitivity to specific environmental parameters. Ecological interactions, nutrient availability, and local environmental conditions (T °C, pH, etc.) are factors likely to influence this dynamic [54,64,76]. Other studies have indicated that water flow [77] and oxygenation [78] contribute to the shaping of microbial communities in biofilms. Combined with temporal heterogeneity, geographic heterogeneity was observed with genera present in higher proportions at some sites than at others, in agreement with previous observations [79,80].

Statistical analysis of alpha diversity showed significant differences among sites (*p* = 0.03), indicating variations in species richness and equitability. No significant difference in diversity was observed between sampling dates (*p* = 0.88), suggesting that species diversity remained relatively stable during this time; however, a longer study period would be necessary to reach conclusions about the stability of the microbiota over time. Our results on diversity among the sites are in line with those of Zancarini et al. [81], who observed significant variations in the composition of the microbial community between collection points up and down a 122 km river. The variations were attributed to differences in water temperature, with an average difference of 5 °C between upstream and downstream sites, and also in the geological and hydrological characteristics of the sampling sites [81]. In our study, no significant difference was found in beta diversity between sites or sampling dates, although subtle variations in bacterial community structure were observed between sampling dates (*p* = 0.07). These observations suggest subtle changes in the composition of bacterial communities over time, which could be linked to environmental factors such as water temperature [82,83,84] or to events that influence the structure of microbial biofilms in freshwater samples. These results contrast with those of two previous studies, in which significant differences were observed between sampling sites [81,85] but not between sampling dates [81], which suggests that the composition of the microbial community is relatively stable over time but may vary according to the characteristics of each sampling site [81,84]. Other studies have also shown that the composition of phyla within biofilms can be influenced by pollution, seasonal variations and the physico-chemical properties of the water [54,64,76,86]. These observations highlight the sensitivity of biofilm communities not only to geographical location, and thus the environment, but also to time, particularly seasons [82,84,87].

## 4. Materials and Methods

### 4.1. Sampling of River Water and Biofilms

**Figure 5:**
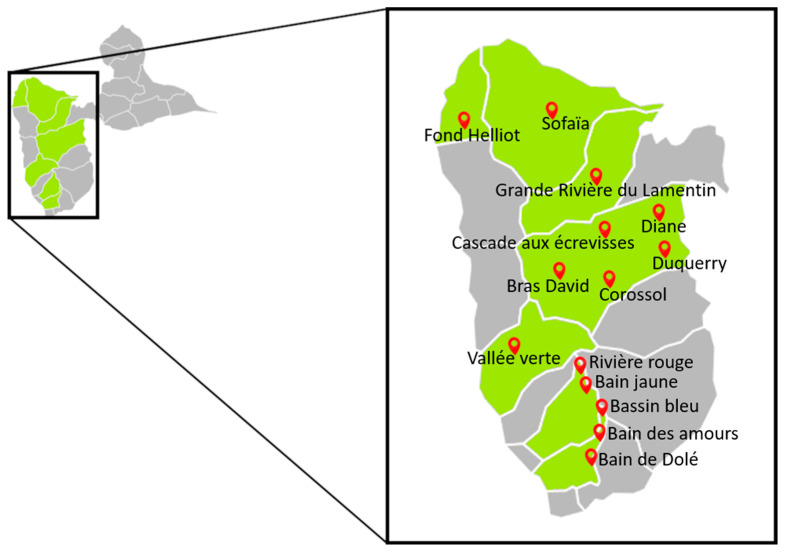
Map of Guadeloupe and location of the sampling sites in the seven municipalities (in green) in the southern part of the Island.

Five sampling campaigns to collect river water were carried out between March and June 2022, coinciding with the dry season. Fourteen rivers in seven municipalities were selected in 2021 on the basis of data from annual monitoring of bathing water quality by the Regional Health Agency for frequent poor water quality (100 > *E. coli* CFU/100 mL ≥ 1800). Water samples were taken approximately 30 cm below the surface in a sterile container, always at the same GPS point. All the rivers are located on the island of Basse-Terre and are in the same hydro-ecoregion, a homogeneous zone with respect to geology, climate, and landscape (Figure 5). Each site was sampled at least three times during the five campaigns.

Two 3-week campaigns to collect biofilms were conducted in May and June 2022. The three rivers selected because of their average or poor water quality were Grande Rivière du Lamentin (16.22603° N; 61.66128° W), Corossol River (16.17071° N; 61.68838° W), and Fond Helliot River (16.28408° N; 61.79241° W).

A total of fifty-nine river water samples and six biofilm samples were taken for bacteriological analysis. All samples were placed in a refrigerated container (4 °C) during transport and protected from sunlight until their delivery to the laboratory. The samples were then kept in a refrigerator at 4 °C and analysed within 24 h.

### 4.2. Enumeration of Escherichia coli in Water

The MPN of *E. coli* was determined on MUG microplates (Bio-Rad, Marnes-la-Coquette, France) according to NF EN ISO 9308-3:1998 [88]. To a tube containing 18 mL of special microplate diluent, 18 mL of sample was added to obtain a 1:2 dilution. The tube was vortexed and 2 mL of the suspension was removed and added to another tube containing 18 mL of special microplate diluent to obtain a final dilution of 1:20. For enumeration of third-generation cephalosporin-resistant *E. coli*, 1 mL of ceftriaxone antibiotic solution was added to the dilutions for a final concentration of 4 mg/L.

For each river water sample, a microplate containing dehydrated *E. coli*-specific culture medium was halved. The 1:2 dilution was distributed into 64 wells and the 1:20 dilution into the remaining 32 wells with an eight-channel pipette set at 200 µL. Plates were covered with sterile adhesive strips and incubated at 44 °C for 36–72 h, and the number of positive wells was converted into the MPN from the MPN table. The values obtained were compared with the standard threshold values provided in the European Directive of 15 February 2006 (2006/7/EC) [89] and the Public Health Code: Articles L.1332-1 to L.1332-9 and D.1332-14 [90,91].

### 4.3. Isolation, Identification, and Susceptibility of E. coli in Water

*E. coli* strains were isolated by the membrane filtration method as described previously [19]. Filters and positive wells were inoculated onto chromogenic coliform agar medium either without antibiotics or supplemented with ceftriaxone at 4 mg/L. After incubation for 24 h at 37 °C, 10 dark blue CFUs (presumptive *E. coli* onto chromogenic coliform agar) were plated on non-selective bromo-cresol purple agar. Blue presumed *E. coli* colonies were identified by matrix-assisted laser desorption/ionisation mass spectrometry, and colonies of other colours were selected at random for this exploratory research. After species confirmation, a maximum of three *E. coli* colonies was collected from media on which growth was observed, for a total of 152 *E. coli* isolates. One strain per sample (*n* = 47) from the agars on which bacterial growth had been observed was chosen arbitrarily for analysis of antibiotic resistance phenotypes. The disc diffusion technique on Mueller–Hinton agar was used according to the recommendations of the Antibiogram Committee of the French Society of Microbiology [92] (http://www.sfm-microbiologie.org, accessed on 18 July 2023).

The strains were tested against a panel of 16 antimicrobial agents corresponding to those most commonly prescribed in human and veterinary medicine: amikacin (30 µg), ciprofloxacin (5 µg), amoxicillin/clavulanic acid (20 μg/10 μg), ampicillin (10 µg), cefoxitin (30 µg), ceftazidime (10 µg), cefotaxime (5 µg), ertapenem (10 µg), fosfomycin (200 µg), gentamicin (10 µg), nalidixic acid (30 µg), streptomycin (10 µg), temocillin (30 µg), tetracycline (30 µg), ticarcillin (75 µg), and trimethoprim/sulfamethoxazole (25 µg). Growth inhibition diameters were measured with the Adagio automated system (Bio-Rad). The critical diameter reference proposed by CA-SFM/EUCAST 2021 [92] was used to interpret the diameter of the zone of inhibition. Isolates that showed a resistant or intermediate phenotype were classified as resistant strains. *E. coli* ATCC 25,922 was used as the control strain.

### 4.4. Biofilm Collection, DNA Extraction, and Resistome Analysis

Five rocks per river site were collected and identified. After sterilisation, the rocks were placed in a net and deposited in the river at a location with little current. After 21 days of immersion, to allow for natural biofilm formation with the microbial communities of the river, the rocks were collected and carefully scraped with a sterile toothbrush, which was regularly rinsed with a small volume of sterile water [84,85]. The solution obtained was then centrifuged at 8000× *g* for 10 min to remove excess water, and the resulting pellet was stored at −80 °C until analysis.

Water samples were collected at the same time as rocks to ensure monitoring of *E. coli* contamination rates at the sites.

Bacterial DNA from biofilms was extracted with a NucleoSpin Soil kit (Macherey—Nagel GmbH & Co. KG, Düren, Germany) according to the manufacturer’s protocol. DNA concentrations were measured with the Qubit instrument (Thermo Fisher Scientific, Waltham, MA USA). Some DNA from each sample was sent to the Biomics Platform C2RT (Institut Pasteur, Paris, France) for microbial community analysis and the remaining DNA was sent to the University of Limoges for the analysis of biofilm resistomes. The method used for resistome analysis was 96.96 BioMark^®^ Dynamic Array for Real-Time PCR (Fluidigm Corporation, San Francisco, CA, USA). Threshold cycle (Ct) values were extracted with BioMark Real-Time PCR analysis software v1.2.0. Normalised gene abundance was calculated from 16S rRNA gene abundance according to the following formula: 2^{−[Ct(RG) − Ct(16S rRNA)]}.^ The analytical procedure is described in more detail by Buelow [93,94].

### 4.5. Molecular Identification of Bacteria by 16S rRNA Gene Sequencing and Metagenomic Analysis

Amplification of the V3–V4 region of the 16S rRNA gene and MiSeq sequencing was performed by the Biomics platform of the Institut Pasteur Paris, France.

The V3–V4 variable region of the ribosomal DNA coding gene was amplified with the following prokaryote-specific universal primers: 341F (CCTACGGGNGGCWGCAG) and 785R (GACTACHVGGGTATCTAATCC) [95]. After Illumina sequencing, the raw data were cleaned with the “dada2” library [96] for use in R software v4.3.0 [97], which allows for filtering, merging, clustering, chimera removal, and taxonomy assignment (on the SILVA database [98]) of the amplicon sequence variants found. Data were analysed with “ggplot2” [99] and “phyloseq” [100] in order to generate diagrams according to the desired conditions. Statistical analyses were performed with the “vegan” library [101].

## 5. Conclusions

In Guadeloupe, rivers not affected by WWTP discharges have relatively low levels of ARB. Nevertheless, the frequent presence of *E. coli* in recreational freshwater, sometimes at concentrations that exceed regulatory standards, raises concern about the health of bathers. The analysis of bacterial communities at various sites showed similar compositions but different abundances, reflecting site-specific environmental conditions. The analysis of biofilm resistomes revealed traces of MGEs and ARGs, confirming limited antibiotic resistance in environments that do not receive human discharge, although clinical ARGs were identified. Better management of hot spots, such as those that receive effluents from WWTPs, tourist areas, and hospitals, should prevent the contamination of recreational areas by faecal bacteria and, consequently, help avoid the risk of dissemination of bacterial resistance.

## Figures and Tables

**Table 1:** Numbers of *E. coli* (most probable number, CFU/100 mL) in river water in Guadeloupe.

		Most Probable Number (CFU/100 mL)				
Municipality	Site	Campaign 1	Campaign 2	Campaign 3	Campaign 4	Campaign 5
Deshaies	Fond Helliot	30	49	144	77	347
Gourbeyre	Bain des Amours	0	15	61	61	30
Gourbeyre	Bassin Bleu	–	75	61	15	46
Gourbeyre	Bain de Dole	46	0	46	30	160
Lamentin	Grande Rivière du Lamentin	110	2213	179	144	127
Petit Bourg	Bras David	15	15	1336	15	–
Petit Bourg	Cascade aux Ecrevisses	15	15	30	46	61
Petit Bourg	Corossol	0	–	2508	0	46
Petit Bourg	Diane	15	46	77	77	–
Petit Bourg	Duquerry	0	15	15	15	–
St Claude	Bain Jaune	–	–	30	0	0
St Claude	Rivière Rouge	–	15	94	0	0
St Rose	Sofaïa	–	0	–	0	0
Vieux Habitants	Vallée Verte	–	0	1071	0	0

– Not collected.

## Data Availability

Data are contained within the article.
